# Editorial: Neuropsychological testing: from psychometrics to clinical neuropsychology

**DOI:** 10.3389/fpsyg.2025.1549236

**Published:** 2025-01-28

**Authors:** Alessio Facchin, Elisa Cavicchiolo, Edgar Chan

**Affiliations:** ^1^Neuroscience Research Center, Department of Medical and Surgical Sciences, Magna Graecia University, Catanzaro, Italy; ^2^Department of Systems Medicine, University of Rome Tor Vergata, Rome, Italy; ^3^Department of Neuropsychology, The National Hospital for Neurology and Neurosurgery, London, United Kingdom

**Keywords:** psychometrics, testing, test development research, reliability, validity

Neuropsychological testing represents an essential part of the clinical examination of neurological patients, and these measures remain the primary instrument for clinical research in neuropsychology (Bauer et al., 2012; Bondi and Smith, 2014; Howieson, 2019). It is crucial that neuropsychological tests are regularly reviewed and updated in order to remain relevant and useful. New research is needed to improve neuropsychological testing as well as help understand the psychometric characteristics and theories behind the tests we use (Bilder and Reise, 2019; Casaletto and Heaton, 2017; Randolph, 2002). This Research Topic on “*Neuropsychological Testing: From Psychometrics to Clinical Neuropsychology*” brings together a collection of articles that examine recent developments in test development and validation across a range of cognitive domains and clinical settings. The emerging picture underlines the complexity of bridging clinical needs with basic psychometric research.

## New test development in emerging areas

The development of novel neuropsychological tests is crucial to advance our understanding of brain-behavior relationships in the ever changing social context. Innovative testing methods which incorporate new technology or advances in cognitive neuroscience allow us to better capture cognitive changes and provide more personalized treatment plans (Parsons and Duffield, 2020). As the field of neuropsychology and neurorehabilitation moves toward a greater dependence on computerized or digitalized tools, it is important to consider the suitability of these tools for the individual.

The article (Stoll et al.) explores this concept using the “Digital Tools Test” (DIGI), a standardized instrument designed to evaluate digital tool competencies in a sample of young people and older adults. Preliminary results highlight performance differences between age groups, with older adults showing lower proficiency in navigating digital tools. In the future, digital tool competency assessments like the DIGI may be used in standard neuropsychological assessments. As technological advances allow for biometric measurements to be more accessible, the study (Gomes et al.) explores the use of both response type/time and eye-fixation measures to detect feigned memory impairment through a computerized version of the well-established Test of Memory Malingering (TOMM). Results found distinct behavioral patterns for genuine and feigned memory impairment. The findings highlight the potential of how eye-tracking metrics may enhance standard paper-and-pencil neuropsychological tools. Finally, the opinion piece (Finley) discusses the use of digital technologies to enhance Performance Validity Assessment (PVA).

Taking an alternative approach, the article (Elkana) explores the “frontal lobe paradox” by discussing the importance of using Real-Life Tasks (RLTs) to enhance standard paper-and-pencil tasks. The “frontal lobe paradox” is a well-described phenomena in neuropsychology whereby some patients with frontal lobe compromise report a host of executive difficulties in daily activities but perform reasonably well in standardized neuropsychological tests. A framework for assessing frontal dysfunction using a variety of RLTs is presented.

## Psychometric evaluation or validation

The evaluation of psychometric properties is essential for selecting reliable and valid instruments, making it a fundamental aspect of clinical practice and research in many areas (Souza et al., 2017). Unfortunately, many instruments still lack thorough or complete validation, which hinders their practical application (Monticone et al., 2021). In this Research Topic, particular emphasis has been placed on the psychometric properties of various existing neuropsychological instruments, and notable advancements have also been reported.

The study (de Oliveira et al.) presents the development and initial validation of a new tool for the Assessment of Reading and Executive Functions (AREF) in children. The findings highlight the interdependence of executive functions, such as inhibitory control, cognitive flexibility and working memory, with reading skills. Once new tests such as the AREF are validated and in use, further validation studies and developments can improve its clinical utility. Country-specific validation of tests is useful to overcome inherent cultural, language and educational differences. The study (Taroza et al.) investigated the psychometric properties of the EQ-5D-5L instrument for assessing health-related quality of life (HRQoL) in Lithuanian individuals who have experienced stroke, while the study (Shi and Zhang) investigated the reliability and validity of the Broken ring enVision search (BReViS) test for assessing attention in the Chinese population.

It is also important to understand the test-retest reliability of our tools for monitoring change over time. The study (Isernia et al.) investigates the test-retest reliability of the Yoni-48 task, a tool for assessing Theory of Mind (ToM) in social cognition, and to establish the minimal detectable change for determining clinical significance. Lastly, shortening established tests can often improve clinical utility but it is important that the same validation rigor is applied before use. The study (De Luca et al.) focuses on the development of the Short Italian Wilkins Rate of Reading Test to enhance the test's applicability to elderly and neuropsychological patients by reducing reading time compared to the original standard form.

## Reviews

Meta-analysis and systematic reviews provide a comprehensive understanding of test properties by synthesizing vast amounts of research on a given topic. These studies help ascertain clinical utility with greater power and guide future research. The article (Malek-Ahmadi and Nikkhahmanesh) presents a systematic review assessing the diagnostic accuracy of the Montreal Cognitive Assessment (MoCA) for detecting amnestic mild cognitive impairment. The findings support the MoCA's utility as a screening tool in clinical settings but emphasizes the need for context-specific cutoff adjustments. The article (Maiuolo et al.) provides a critical evaluation of the scale used to assess wellbeing in people with Parkinsonism. Although eight HRQoL tools were identified, questions were raised about the psychometric properties of the measures which may mar their utility.

## Summary

Articles in the Topic highlight the interplay between psychometrics and Clinical Neuropsychology. Continued research into novel measures, applications, comparisons and updates is crucial for maintaining and improving the clinical practice of neuropsychological testing.
